# Robotic versus hand-assisted laparoscopic living donor nephrectomy: comparison of two minimally invasive techniques in kidney transplantation

**DOI:** 10.1007/s11701-022-01393-x

**Published:** 2022-03-07

**Authors:** Olivier Laurent Windisch, Maurice Matter, Manuel Pascual, Pamela Sun, Daniel Benamran, Leo Bühler, Christophe Emmanuel Iselin

**Affiliations:** 1 Geneva-Lausanne Transplant Center (Centre Universitaire Romand de Transplantation), Hôpitaux Universitaires de Genève, Rue Gabrielle-Perret-Gentil 4, 1205 Genève, Switzerland; 2 Division of Urologic Surgery, Geneva University Hospital, Genève, Switzerland; 3 Department of Visceral Surgery, Lausanne University Hospital, and University of Lausanne, Lausannne, Switzerland; 4 Transplantation Center, Lausanne University Hospital, and University of Lausanne, Lausannne, Switzerland; 5 Section of Medicine, Faculty of Science and Medicine, University of Fribourg, Fribourg, Switzerland

**Keywords:** Robotic surgery, Living donor nephrectomy, Minimally invasive surgery, Hand-assisted laparoscopic nephrectomy, Robotic donor nephrectomy, Warm ischemia time

## Abstract

Robot-assisted donor nephrectomy (RDN) is increasingly used due to its advantages such as its precision and reduced learning curve when compared to laparoscopic techniques. Concerns remain among surgeons regarding possible longer warm ischemia time. This study aimed to compare patients undergoing robotic living donor nephrectomy to the more frequently used hand-assisted laparoscopic nephrectomy (HLDN) technique, focusing on warm ischemia time, total operative time, learning curve, hospital length of stay, donor renal function and post-operative complications. Retrospective study comparing RDN to HLDN in a collaborative transplant network. 176 patients were included, 72 in RDN and 104 in HLDN. Left-sided nephrectomy was favored in RDN (82% vs 52%, *p* < 0.01). Operative time was longer in RDN (287 vs 160 min; *p* < 0.01), while warm ischemia time was similar (221 vs 213 secs, *p* = 0.446). The hospital stay was shorter in RDN (3.9 vs 5.7 days, *p* < 0.01).Concerning renal function, a slightpersistent increase of 7% of the creatinine ratio was observed in the RDN compared to the HLDN group (1.56 vs 1.44 at 1-month checkup, *p* < 0.01). The results show that RDN appears safe and efficient in comparison to the gold-standard HLDN technique. Warm ischemia time was similar for both techniques, whereas RDN operative time was longer. Patients undergoing RDN had a shorter hospital stay, this being possibly mitigated by differences in center release criteria. Donor renal function needs to be assessed on a longer-term basis for both techniques.

## Introduction

Living donor nephrectomy (LDN) is an increasingly used procedure that allows preemptive and timely renal transplantation with optimal clinical outcomes for patients with end-stage renal failure (ESRF). The increase in ESRF is estimated to be at 5% per year in the USA, with a prevalence rising from 300,000 to 700,000 from 1996 to 2015 [1]. Due to this steady increase in patients awaiting transplantation, organ demand raises each year. Since 2009, over 27 000 living donor nephrectomies were performed yearly worldwide, with a majority of countries reporting a 50% increase in the past decade [2]. The Swiss national database reported a 44% increase of patients awaiting renal transplantation from 2008 to 2020 (758 to 1094) while live donor transplantation represented 35% of the transplanted kidneys during this period (1406 from living donor, 2586 from deceased donor) [3]. Due to this increased demand for organs, criteria for donation have been widened, and older patients, as well as patients with relative contra-indications (obesity, prediabetes, kidney stone, or hypertension), have progressively been considered eligible for organ donation under certain conditions [4, 5].

Safety and quality of life of donors are of paramount importance, and donors are now considered to have the same life expectancy and quality of life as non-donors [4]. Since its beginning in 1954, LDN has considerably evolved given increasing donor quality of life and safety. In 1995, Ratner performed the first clinically successful laparoscopic donor nephrectomy and showed a reduction in hospital stay, postoperative pain, and return to normal activity compared to the conventional open technique [6]. Since, multiple minimally invasive techniques have been developed. A meta-analysis on minimally invasive techniques for LDN published in 2016 on more than 32 000 patients showed predominant use of laparoscopic surgery (57.4%) and HLDN (25.3%), while robot-assisted donor nephrectomy (RDN) represented only 1.3% of the procedures [7]. A recent American study on 1084 patients, with a high percentage of obese (39.4%) and overweight patients (34.1%) showed the safety of RDN also for higher BMI patients, with 2.1% Grade III or Grade IV complications according to Clavien–Dindo classification [8].

Robotic surgery is increasingly used in urology and organ transplantation. RDN was first described in 2001 and its technique standardized in 2004 [9, 10]. Since, RDN is increasingly used, and many authors have integrated RDN to their minimally invasive techniques [11]. However, RDN is still a matter of debate among surgeons. Proponents for RDN use the argument of safety and faster recovery for the RDN approach, based on increased maneuverability and control allowing to achieve safely difficult steps of the procedures. Another argument is a shorter learning curve compared to the laparoscopic procedure [11, 12]. Surgeons who oppose RDN are mainly concerned by the warm ischemia time, which is generally longer for RDN than LDN, and state that highly skilled surgeons can usually perform all steps laparoscopically. They also argue potential increased medical costs [13].

In view of the currently ongoing debate, we decided to compare parameters of safety, peri- and postoperative outcomes for robotic surgery and hand-assisted laparoscopic, performed within the Geneva-Lausanne Transplant Center, a collaborative transplant network.

## Materials and methods

This was a retrospective study in kidney transplantation conducted on donors undergoing minimally invasive living donor nephrectomy at two institutions part of the Geneva-Lausanne Transplant Center; Geneva University Hospital (HUG) and Lausanne University Hospital (CHUV). Patients were included between December 1st, 2013 and February 28th, 2019. Only patients who underwent RDN at HUG and HLDN at CHUV were included in the study. The exclusion criterion was explicit refusal to participate in the study. The study was started after approval from the Ethics Commissions; CCER Genève for HUG, CER-VD for CHUV under national registration name: 2020-01454. The inclusion criterion was live-donor voluntary kidney donor, approved by institutional medical, ethical and legal boards for transplantation.

Preoperative workup consisted of an assessment of the renal function by CKD-EPI formula based on baseline creatinine dosage or ^99m^TC-MAG3-scan to assess glomerular filtration rate. All patients underwent preoperative DMSA-scan to assess proportional kidney function. The kidney was considered dominant when contributing more than 55% to the global function. An injected computerized tomography scan allowed to assess vascularization. Kidney side choice was based on classic criteria in the following order when possible; dominant kidney left to the donor, vascular anatomy, and others (kidney malformation, urolithiasis, renal cysts). All patients underwent presentation to a multidisciplinary transplantation board after the side was chosen to confirm eligibility and rationale of the side choice. Each institution’s ethical board validated the donor eligibility.

At HUG, HLDN and retroperitoneoscopic living donor nephrectomy were performed from 2003 to December 2013, when RDN was initiated as part of the living kidney donor program. Standard LDN (Rattner, 1995) was introduced at CHUV in 1998, and laparoscopic HLDN in 2005 and is still the usual practice at CHUV. Hence the opportunity was given to assess the role of robot-assisted surgery in comparison to the technique performed on a routine national standard basis in Switzerland. The RDN technique was performed by a team composed of four surgeons at HUG; a senior surgeon with extensive experience in laparoscopic and robotic surgery, and 3 fellow surgeons used to laparoscopic surgery and training in robotic surgery. Of the 3 fellows, only one performed consequent caseload, which was analyzed in the teaching analysis. Regarding teaching analysis, cases (*n* = 5) performed by the other fellows were excluded. The dual console was used for every teaching case. The HLDN technique was performed by a team of two surgeons at CHUV; one experienced surgeon and a fellow surgeon. Teaching was retrospectively assessed, and defined as partial (steps of the interventions performed by the trainee surgeon) or complete (all steps completed by the trainee surgeon).

Data regarding donor demographics, operative and post-operative details were collected from both computerized institutional medical record systems. Operative details included kidney laterality, total duration of operation from initial incision to skin closure, warm ischemia time, intraoperative teaching. Post-operative details included creatinine at discharge, creatinine at 1 month, total length of stay, post-operative complications scored according to the Clavien–Dindo classification [14]. After extraction, all data were coded and anonymously analyzed.

### Surgical techniques protocols

#### Robot-assisted laparoscopic surgery

**Fig. 1:**
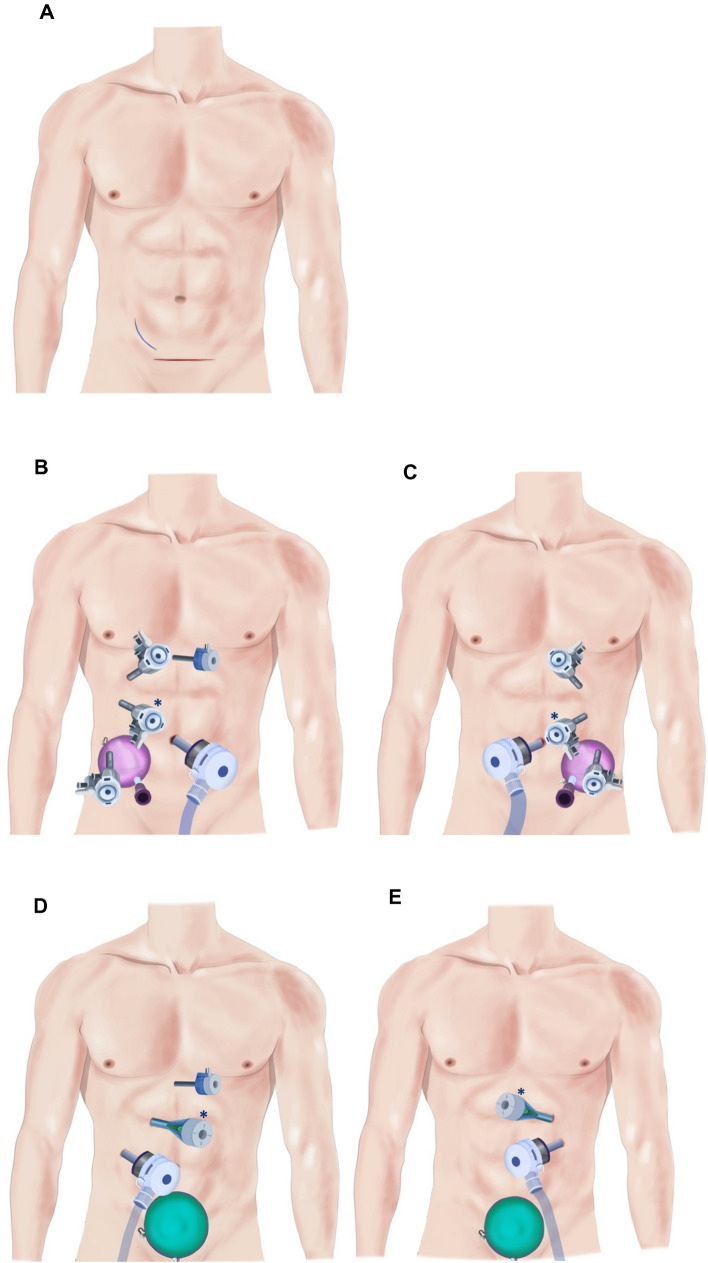
Incision and trocar placements in RDN and HLDN. A: Skin incision for right nephrectomy (RDN: 7–8 cm flank incision, HLDN: 10 cm Pfannenstiel incision). B: Right RDN. C: Left RDN. D: Right HLDN. E: Left HLDN. *: Camera (RDN: 8 mm for Xi robot, 12 mm for Si robot, HLDN: 11 mm). For RDN, the trocars inside the Gelport/GelPOINT are 3 finger breadths apart. RDN, Robot-assisted donor nephrectomy, HLDN**,** Hand-assisted laparoscopic nephrectomy

The Da Vinci Si Robot was used until April 2015 for the first 21 cases, and changed for the Xi version afterwards. The patient was installed in a modified contralateral decubitus position, with the hip flexed to the back at a 30° angle. First, a 12 mm incision was made at the umbilicus and a Veress needle inserted, creating a pneumoperitoneum of 12 mmHg. After the withdrawal of the Veress needle, an Airseal^®^ 12 mm trocar was then inserted at the umbilicus, followed by a subcostal da Vinci trocar on the mid-clavicular line. A second da Vinci trocar was placed at the level of the umbilicus between the medioclavicular and axillary line (camera, 12 mm Si, 8 mm Xi). An oblique mini-laparotomy of 7–8 cm in the ipsilateral iliac fossa was performed to simultaneously prepare for kidney extraction so as to be used for placement of two further ports. Access to the peritoneum was obtained by medializing the rectus abdominis muscle and rarely, when required, minimally incising (0–2 cm) laterally the oblique musculature. A Gelport^®^ or GelPOINT^®^ device was then placed, while 8 mm da Vinci and 10 mm assistant trocars were inserted through the device, 3 finger breadths apart; see Fig. 1. For right nephrectomy, another retraction trocar was inserted under the xiphoid appendix to recline the liver cephalad. Docking of the robotic arms was then performed and the rest of the procedure followed standard steps, briefly exposed here since well standardized in current literature: colic medialization, en bloc gonado-ureteral dissection starting at the iliac vessels, hilar venous and arterial dissection, and kidney mobilization. After ureter section at the iliac vessels and administering iv 5000 U of heparin, artery and vein were doubly clamped with Hem-o-Lok^®^ clips and sectioned. Dedocking of the iliac arm of the robot was performed and the harvested kidney was then seized on its inferior pole with an atraumatic grasper inserted through the da Vinci iliac trocar. Consecutively the Gelport^®^ arm was opened and the kidney extracted and transferred to the transplantation team for immediate cold perfusion. Warm ischemia time was measured precisely from vessel clamping to back table observation of active venous efflux. After redocking the da Vinci arm, the arterial stump was secured with a 5.0 Prolene running suture, and hemostasis controlled, followed by dedocking of the robot and closure according to the usual standardized technique.

#### Laparoscopic hand-assisted surgery

The patient was installed in a contralateral decubitus position on a ball mattress, with the table broken at a 30° angle. A Pfannenstiel incision was performed first (sometimes using a previous one in women), the Gelport^®^ was inserted and the pneumoperitoneum initiated (12 mmHg). The hand was introduced and secured the placement of one 12 mm trocar in the ipsilateral iliac fossa (for dissection and staplers), then an 11 mm trocar was inserted under direct vision in the ipsilateral hypochondrium (for the laparoscope). On the right side, an additional epigastric 5 mm trocar helped to retract the liver; see Fig. 1. The rest of the procedure was similar to the abovementioned. Initially, 5000 U of heparin iv. was administered and controlled after the section of vessels by Protamin, but it was progressively abandoned without any consequence regarding morbidity. The artery was first double stapled and cut (Covidien Endo GIA™ Ultra 30 mm. vascular articulated Tri-staple™) and the vein had a simple stapling (Covidien EndoTA 30 mm. Auto Suture™ 30 mm.) and was then cut, to preserve a maximum length. The kidney was removed by hand through the Gelport^®^ and transferred for immediate cold perfusion. Warm ischemia time was measured precisely from vessel clamping to back table IGL-1 cooling and venous efflux. After controlling hemostasis, all incisions were closed according to the usual standardized technique.

Hospital stay was calculated using the day of surgery as day 0. Hospital discharge was not based on medical considerations but at the discretion of the donor (usually between postoperative day 3 until 7), to improve the comfort and relationship with the recipient.

### Statistical analysis

Statistical analyses were conducted using STATA 16, StataCorp. LCC. The Pearson Chi^2^ test or Fisher’s exact test were used for categorical variables when appropriate. Student t-test was used for continuous variables after graphical and descriptive assessment of normal variance. Significance statements refer to *p* values < 0.05 in two-tailed test. To avoid obtaining statistically significant values from test repetitions, the Bonferroni method was used for subgroup analysis to adjust statistical significance threshold. Univariate and multivariate linear regression models were used using all clinically significant variables to assess a correlation between pertinent clinical variables and operative time as well as warm ischemia time. Application conditions were tested (normality of the residues, residues centered on 0, and homoscedasticity of the residues, which was graphically assessed), and models are presented only if those conditions were fulfilled. Because one model could not fit both techniques when testing application conditions, two distinct models, each one by technique, were performed. Only statistically significant variables (*p* < 0.05) were included in the multivariable linear model.

## Results

One hundred-seventy-six patients were included in the study, 72 in the RDN group and 104 in the HLDN group. Baseline donor demographics were comparable in both groups for age, BMI, while female donors dominated in both groups (69% vs 62%) (Table 1). Baseline donor creatinine was similar between both groups.

**Table 1:** Patients demographics and intraoperative data

Patients demographics	HLDN (*n* = 104)	RDN (*n* = 72)	*P* value	Significance threshold*
Age (years)	54.1 (11)	51.3 (11)	0.124	
Gender (no female, % female)	70 (67)	50 (69)	0.765	
BMI (kg/m^2^)	25.2 (4)	24.9 (3)	0.638	
Baseline creatinine (µmol/l)	72.9 (12)	70.4 (13)	0.212	
Kidney side				
Left	54 (52)	59 (82)	0.01	
Reason for kidney side selection			0.015	
Longer vein (identical kidneys)	32 (31)	34 (47)		
Vascular reason (multiples arteries or veins)	44 (42)	32 (45)		
Asymmetrical kidney function (better kidney left to the donor)	23 (22)	5 (7)		
Other (cysts, kidney stones, Nutcracker syndrome)	4 (4)	1 (1)		
Vascular and better function	1 (1)	0		
Vascular anatomy				
Double arteries	17 (16)	12 (17)	0.955	
Double veins	10 (10)	5 (7)	0.533	
Operative time (minutes)				
Global	160 (39)	287 (44)	< 0.001	
Left side	170 (44)	273 (36)	< 0.001	0.006*
Right side	149 (30)	290 (45)	< 0.001	0.006*
Partial teaching	176 (32)	287 (24)	< 0.001	0.006*
Complete teaching	237 (20)	283 (30)	0.008	0.006*
Single artery	159 (38)	283 (42)	< 0.001	0.006*
Double artery	163 (43)	307 (47)	< 0.001	0.006*
Single vein	162 (39)	286 (43)	< 0.001	0.006*
Double vein	143 (40)	305 (50)	< 0.001	0.006*
Warm ischemia time (seconds)				
Global	213 (77)	221 (57)	0.47	
Left side	216 (78)	222 (55)	0.65	0.006*
Right side	210 (77)	218 (68)	0.75	0.006*
Partial teaching	203 (75)	225 (45)	0.27	0.006*
Complete teaching	221 (82)	219 (42)	0.93	0.006*
Single artery	197 (64)	217 (56)	0.05	0.006*
Double artery	296 (89)	241 (59)	0.07	0.006*
Single vein	206 (75)	218 (56)	0.31	0.006*
Double vein	276 (24)	268 (26)	0.83	0.006*

Categorical data are presented as absolute numbers (percentage). Quantitative data are presented with mean (standard deviation). All tests are 2-sided. Operative time was defined from skin incision to skin closure and is presented in subgroups according to kidney laterality and presence of teaching. Same subgroup analysis is shown for warm ischemia time

*HLDN* laparoscopic hand-assisted surgery. *RDN* robot-assisted donor nephrectomy

*Adjusted *p* value for significance threshold is displayed using Bonferroni method, when subgroup analysis were performed. When not indicated, usual threshold of *p* = 0.05 was considered significant

All donors in both groups were operated on without the need for conversion to open surgery. Left-sided nephrectomy was favored in the robotic group vs the HLDN group (82 vs 52%, *p* < 0.01). The reason for kidney choice and operative data details are displayed in Table 1. In the RDN group, one donor had the better kidney taken (left, assuming 57% of the global function), because of a simultaneous Nutcracker syndrome. Anatomic complexity (defined by multiple venous or arterial systems) did not differ between the two groups. A longer total operative time was seen in the RDN group compared to the HLDN group (287 min vs 160, *p* < 0.01). The warm ischemia time was comparable in both groups (221 vs 213; *p* = 0.45), without statistical difference between kidney side.

Surgical teaching was performed in both institutions, representing 44% of the HLDN technique and 33% of the RDN technique (*p* = 0.15). The first complete intervention by the trainee surgeon was performed after 37 procedures in the HLDN technique, and 10 in the RDN technique.

Postoperative data are displayed in Table 2. Minor postoperative complications developed in both groups in similar proportions, and major complications (≥ 3a) appeared in both groups in less than 2% of the cases. Two donors had to be reoperated under general anesthesia (Clavien III b), one for a wound abscess and another for extraperitoneal venous bleeding of the Pfannenstiel incision 3 days after surgery in HLDN, while one required percutaneous drainage of an infected chyloperitoneum (Clavien III a) in RDN. The length of stay was significantly shorter in the RDN, with a mean hospital stay of 3.9 days compared to 5.7 in the HLDN group (*p* < 0.01).

**Table 2:** Postoperative period

Postoperative course	HLDN (*n* = 104)	RDN (*n* = 72)	*P* value
Mean length of hospital stay (days)	5.7 (1.9)	3.8 (1.4)	< 0.01
Median length of stay (days)	6 (5–6)	3 (3–4)	< 0.01
Complications (Clavien-Dindo classification)			
No or minor complications (Grade ≤ 2)	95 (98)	71 (99)	0.364
Major complications (Grade ≥ 3	2 (2)	1 (1)	
Complications details (Clavien–Dindo classification)			
0	90 (86)	58 (81)	
I	**5** (5)	**11** (15)	
II	**7** (7)	**2** (3)	
IIIA	0 (0)	1 (1)	
IIIB	2 (2)	0	
IV and +	0	0	

Categorical data are presented as absolute numbers (percentage or interquartile range). Quantitative data are presented with mean (standard deviation). All tests are 2-side

*HLDN* laparoscopic hand-assisted surgery. *RDN* robot-assisted donor nephrectomy

Similar serum creatinine between both groups was observed at baseline, hospital discharge, and 1-month control checkup as shown in Table 3. When the discharge and 1-month postoperative values were compared to baseline values, a persistent increase of 7% of the ratio was observed in the RDN compared to the HLDN group (1.60 vs 1.49 at discharge, *p* < 0.01, 1.56 vs 1.45 at 1-month post-op, *p* < 0.01)(Table 3).

**Table 3:** Kidney function evolution

Kidney function	HLDN (*n* = 104)	RDN (*n* = 72)	*P* value
At baseline			
Creatinine (µmol/l)/(mg/dl)	72.9 (12)/0.83 (0.13)	70.4 (14)/0.80 (0.16)	0.212
At discharge			
Creatinine (µmol/l)/(mg/dl)	108.5 (23)/1.23 (0.26)	111.1 (23)/1.26 (0.26)	0.461
Discharge/baseline creatinine ratio	1.49 (0.2)	1.60 (0.2)	< 0.01
1 month postoperative			
Creatinine (µmol/l)/(mg/dl)	105.3 (20)/1.20 (0.23)	108.3 (21)/1.23 (0.24)	0.17
Discharge/baseline creatinine ratio	1.45 (0.2)	1.56 (0.2)	< 0.01

Quantitative data are presented with mean (standard deviation). All tests are 2-sided

*HLDN* laparoscopic hand-assisted surgery. *RDN* robot-assisted donor nephrectomy

Regression models for total operative time and warm ischemia time are displayed in Tables 4 and 5. Two models were necessary since one model did not fit both techniques. Univariate results are included in Supplementary Table 1 and 2 for readability reasons. In RDN, multivariate analysis showed that total operative time was statistically associated with following characteristics; decreased with intervention number, (− 1.14 min per intervention, *p* < 0.01), increased with partial teaching (+ 36.2 min, *p* = 0.005), even more by complete teaching (+ 46.5 min, *p* = 0.01), double arteries (+ 25.8 min, *p* = 0.03), and male gender (+ 38.4 min, *p* < 0.01). In HLDN, the multivariate analysis showed that the total operative time increased with intervention number (+ 0.31 min per intervention), partial teaching (+ 28.2 min, *p* < 0.01), complete teaching (74.9, *p* < 0.01), BMI (+ 1.22 min per 1 point in BMI increase), left kidney (+ 13.3 min, *p* = 0.02) and male gender (+ 22.9 min, *p* < 0.01). Concerning warm ischemia time, only male gender was significantly associated with longer ischemic time in RDN (+ 37.3 s, *p* = 0.016), whereas complex hilar anatomy appeared as significantly associated with longer warm ischemic time in HLDN; double artery (+ 92.7 s, *p* < 0.01), double vein (+ 67.2 s, *p* < 0.01). Male gender was also associated with longer ischemic time in HLDN (+ 33.7 s, *p* = 0.017).

**Table 4:** Multivariate regression models for variables associated with total operative time

Total operative time	Coefficient (minutes)	HLDN		Coefficient (minutes)	RDN*	
		95% CI	*P* value		95% CI	*P* value
Intervention number	0.313	0.096–0.53	0.005	− 1.14	− 1.7 to 0.60	< 0.001
Teaching						
Partial	28.2	15.05–41.4	< 0.001	36.2	11.1–61.4	0.005
Complete	74.9	46.4–103.4	< 0.001	46.5	10.2–82.9	0.013
BMI	1.22	0.014–2.43	0.047	− 1.51	− 4.49 to 1.47	0.314
Left kidney (vs. right)	13.3	1.98–24.6	0.022	5.17	− 17.9 to 8.3	0.45
Double artery	9.95	− 5.16 to 25.1	0.194	25.8	2.56–49.0	0.03
Double vein	1.00	− 18.0 to 20.0	0.917	10.3	− 24.3 to 44.9	0.554
Male gender	22.9	11.5–34.5	< 0.001	38.4	18.6–58.2	< 0.001
Constant	68.8	32.3–105.4	< 0.001	301	281–321	0.000

Coefficient is expressed in absolute values (minutes) for dichotomous variable, and coefficient for continuous variables. 95% CI refer to the 95% confidence interval of the coefficient. All clinically pertinent variables are included in the model. To ease understanding of the coefficient, an example would be in RDN, a partial teaching on a double artery kidney would add 67.4 min (35.2 + 32.4) compared to no teaching on a single artery kidney operated with the same technique

*HLDN* laparoscopic hand-assisted surgery. *RDN* robot-assisted donor nephrectomy

*Excluding 5 teaching cases operated by 2 other fellows

**Table 5:** Multivariate regression models for variables associated with warm ischemia time

Warm ischemia time	Coefficient (seconds)	HLDN		Coefficient (seconds)	RDN*	
		95% CI	*P* value		95% CI	*P* value
Intervention number	0.122	− 0.40–0.64	0.47	− 0.09	− 0.93 to 0.74	0.826
Teaching						
Partial	4.81	− 26.5–36.1	0.761	23.5	− 14.8 to 61.8	0.224
Complete	4.73	− 63.6–73.1	0.891	10.7	− 44.6 to 66.1	0.700
BMI	2.30	− 0.59–5.19	0.117	1.09	− 3.44 to 5.62	0.632
Left kidney (vs. right)	16.4	− 10.9–43.7	0.235	2.47	− 32.7 to 37.6	0.889
Double artery	92.7	56.4–129.1	< 0.001	23.46	− 11.9–58.8	0.190
Double vein	67.2	21.3–113.1	0.005	42.4	− 10.4 to 95.1	0.114
Male gender	33.7	6.13–61.3	0.017	37.3	7.22–67.5	0.016
Constant	105.7	27.8–183.6	0.008	170.5	48.6–292.3	0.007

Coefficient is expressed in absolute values (minutes) for dichotomous variable, and coefficient for continuous variables. 95% CI refer to the 95% confidence interval of the coefficient. All clinically pertinent variables are included in the model. To ease understanding of the coefficient, an example would be with HLDN, a double artery on a male patient would add 159.9 (92.7 + 67.2) seconds compared to a female patient with a simple artery operated with the same technique

*HLDN* laparoscopic hand-assisted surgery. *RDN* robot-assisted donor nephrectomy

*Excluding 5 teaching cases operated by 2 other fellows

## Discussion

Overall, RDN appeared to be equivalent to the gold-standard HLDN technique in terms of safety, and efficacy. Particularly, warm ischemia time, was comparable between both techniques, while the total operative was longer for RDN. These findings give additional evidence of the feasibility of the RDN technique, especially regarding warm ischemia time which has been the main concern of many surgeons.

Various perspectives may favor either RDN or HLDN technique. Donor security is of utmost importance and has been the driver for constant evolution in the pre-,intra- and postoperative care of live donors. Laparoscopic surgery, with or without hand-assistance has been accepted as the gold standard for living donor nephrectomy, due to its numerous advantages on the postoperative recovery, such as decreased length of stay, use of intravenous analgesics and time to resume normal activity, as well as a better corporeal image for donors [15, 16]. In 2006, Horgan reported the largest cohort of 273 patients who underwent RDN combined with hand-assisted surgery, and confirmed the option of robotic surgery for living donor kidney harvesting [16]. In 2015, Cohen observed a reduced length of hospital stay with RDN compared to HLDN after the learning curve was achieved (1.55 vs 2.00 days) [15]. Despite encouraging reports, literature comparing RDN to other minimally invasive techniques remains scarce and mostly retrospective. A recent meta-analysis showed a mean operative time ranging from 139 to 306 min, ischemia time from 1.5 to 5.8 min and conversion rate between 0 and 5%, with complications ranging from 0 to 16% [17]. Compared to highly trained surgeons in high-volume centers performing robotic and laparoscopic surgery, our findings in the current study lie within normal standards and show the accuracy of robotic surgery in a medium-volume center for robotic procedures.

Robot-assisted surgery appears to be useful in terms of complex dissection, reduced learning curve and shorter hospital stay compared to other laparoscopic techniques. Indeed, the da Vinci robot offers the addition of microsurgical precision to laparoscopic surgery, easier maneuverability of the instruments, as well as better identification of dissection planes [18]. Our study tends to confirm this overall increased maneuverability, by the absence of prolonged warm ischemia in the presence of complex vascular anatomy (multiples arteries or veins) in the RDN group, while double arteries and veins appeared significantly associated with longer warm ischemia time in the HLDN group on multivariate analyses. These findings underline the possible role of RDN for achieving complex hilar dissection, such as retrocaval or inter-aorto-caval right kidney artery dissection for example. Also, although not reaching statistical significance, increasing BMI was associated with decreased operative time, which joins findings of previously published findings in the US cohort [8].

Another advantage of robot-assisted surgery, especially in a living donor program, is the reduced learning curve required to achieve standard results. Since the major issue of laparoscopic surgery is its steep learning curve, laparoscopic living donor nephrectomy requires a high caseload and significant prior laparoscopic experience in kidney and adrenal surgery. Different factors help surgeons to define when the learning curve is achieved; for example, when the surgeon can perform all the steps of the intervention, or when operative time or complication rate reaches a plateau. For kidney donor nephrectomy, the laparoscopic technique is especially challenging because artery and vein length need to be optimal, and multiples vessels, as well as vascular abnormalities, are common. Su published a series of 381 pure laparoscopic live donor nephrectomy patients, of which the first 95 patients had a 21% complication rate compared to the 96 last patients who had a 10% complication rate, suggesting a learning curve threshold around 100 patients [19]. Compared to pure laparoscopic access, HLDN appears to shorten the learning curve, since it helps expose vessels and, using manual lateral kidney traction, increase the vessel length of the harvested kidney. Martin et al. also showed a shorter length of operative time and better graft function after a threshold of 36 HLDN cases. However, they had performed more than 300 laparoscopic renal surgeries before the study period, hence possibly lowering the threshold [20]. Robotic surgery may have a shorter learning curve, which can be as low as 20 patients for an already experienced team [15]. In our study, different findings confirm a reduced learning curve for RDN compared to HLDN. First, complete intervention could be performed after 10 partial teachings in the RDN group, whereas 37 partial teachings were necessary for the HLDN group. We must state here that the senior trainee RDN surgeon was simultaneously trained in robotic partial nephrectomy. Also, total operative time decreased with increasing number of cases (− 1.14 min/intervention) in RDN, while it slightly raised in the HLDN (+ 0.31 min/intervention), which might be partly explained by the slower learning curve in HLDN.

In this study, we observed a significantly longer total operative time in the RDN group. Although acceptable compared to the previously published literature, this finding might be explained by the fact that patient inclusion started when the technique was introduced at HUG, and therefore the learning curve was not reached for the senior surgeon at the beginning of the study, whereas the HLDN technique had been a standard procedure for a long time at CHUV since 2005. Possible other reasons for the longer RDN operative duration are the time for docking and undocking of the robot, so as the higher percentage of left-sided kidney choice in the RDN group, which is usually a longer procedure due to more complex venous affluents, even if right nephrectomy is usually described as more challenging [21]. Finally, the necessity to oversew the renal arterial stump after its double Hem-o-lok^®^ clipping (due to the disclaimer of the manufacturer and strong recommendations in the literature for transfixion technique after several avoidable donor death occurred linked to clip dysfunction) also adds a little time compared to stapling [22, 23]. Also, regarding vessel ligation technique, a 2018 meta-analysis suggested longer vessel length when using clips compared to staplers, with the disadvantage of increased warm ischemia time (55 s), that we did not observe in the current study, and additional blood loss (40 ml) [24]. Interestingly, male gender was associated in both groups with a longer operative time and warm ischemia time, possibly explained by the gender distribution difference of fatty tissue, these being proportionally more important in males intra and retroperitoneally [25].

A shorter hospital stay was observed in the RDN group compared to the HLDN group, with a median difference of 3 days. In accordance, other teams have also outlined a shorter hospital stay with the robotic technique [15]. Although speculative, putative explanations may support this finding. First, absence of distension of the skin incision induced by insertion of the intraperitoneal hand through the Gelport may account for less post-operative pain. Second, the ability to achieve finer dissection planes with the robot, possibly decreasing tissue bleeding, trauma, and inflammation, which may be responsible for less post-operative discomfort in the RDN group. However, these differences in hospital stay have to be considered with caution since they were obtained from two different surgical teams whose hospital discharge criteria may differ.

When compared to HLDN, disadvantages of robotic surgery, include the absence of possible emergency control of bleeding with digital pressure, possible longer warm ischemia time, and additional costs. The main inconvenience and critic for LDN robotic surgery is the possible prolonged ischemia time and longer total operative time [13]. Indeed, since RDN requires undocking of the robotic arm through the Gelport to extract the kidney, prolonged ischemia time might happen [26, 27]. However, in this study, despite longer total operative time, we found comparable warm ischemia times in both groups. This finding shows that this limitation can be reduced to a minimum with a simple extraction method using no bag and an experienced team, even in a mid-volume center. Also, the results show a slight but statistically significant difference in the 1-month postoperative kidney function. The kidney function (creatinine ratio) of robotically harvested kidney was 7% inferior to HLDN at 1 month. This could be due to a longer total operative time, and consequently a longer pneumoperitoneum duration in the RDN group, despite working under standard pneumoperitoneum pressure (12 mmHg) during the whole surgery. These finding raise awareness that even though robotic surgery allows precise dissection and eases technical steps of the intervention, attention should be paid to pneumoperitoneum duration and pressures used. However, these findings do not necessarily translate into worse long-term function recovery, since early and late compensatory changes of the remaining kidney still occur after several years of follow-up [28].

### Study limitations and strengths

Our study presents some limitations. First, it is retrospective which carries inherent bias due to the lack of randomization. Also, due to its retrospective nature, only total operative time was recorded for the RDN group, whereas console time might be a more precise indicator to better understand where the difference in operative time resided and better identify areas of improvement. Second, there is a population asymmetry between both surgical teams, a higher percentage of right-sided nephrectomy was observed in the HLDN group. Right donor nephrectomy has been described as possibly more challenging, but remaining feasible and safe when performed by an experienced team, while left donor nephrectomy is usually favored to achieve longer venous length [21, 29]. Despite that venous collaterals are more complex on the left side (gonadal, adrenal, and lumbar) and might partly explain a longer RDN total operative time, this population difference does not appear to change the message of the study, which confirms the safety of both techniques, and gives further evidence of reduced learning curve with the RDN technique. Third, Comparison of hospital stay may also be biased by institutional differences of hospital discharge criteria. Also, differences in ligation devices (surgical clips in the RDN versus surgical stapler in the HLDN group) may explain why double arteries and veins were associated with longer warm ischemia time in the HLDN and not in the RDN group. Also, the current study focused on the feasibility and safety of the procedure on the donor population and did not study the receiver population, so surgical issues related to vessel length or postoperative kidney graft function were not assessed. This underlines that further prospective studies focused on receivers and technical difficulties are required in the field of robotic donor nephrectomy. In this respect, time to implantation appears to be an interesting factor to evaluate in upcoming studies, since literature regarding the effect of warm ischemia time on kidney function is scarce. Different studies showed that longer warm ischemia time was associated with increased risk of delayed graft function [30, 31]. Other studies, among which a large series on 640 patients showed no correlation between warm ischemia time (ranging between 35 and 720 s) and kidney dysfunction [32, 33]. Interestingly, a more recent study suggested that time to implantation exceeding one hour is associated with increased early poor graft function (up to 36% of patients), despite having no effects on long term function [34]. These findings suggest that time to implantation, which takes into account technical difficulties in performing the anastomosis, might be another good indicator of early graft function and a better assessment of sufficient vessel length.

The strength of this relatively large study performed within our common transplant network is to put in perspective some potential advantages of RDN when compared to one of the current gold-standard techniques.

## Conclusion

The results show that RDN appears safe and efficacious in comparison to the current gold-standard HDLN technique. Warm ischemia time was equivalent for both techniques, although operative time was longer in the RDN group. Overall complication rates were low in both groups. The learning curve and hospital stay appeared reduced for the RDN group compared to HLDN, with a caveat regarding the latter, possibly impacted by team-related hospital discharge criteria. Overall, the data show that robotic kidney harvesting is progressively gaining credibility. Further prospective studies, targeted on receiver kidney function and time to implantation, accounting for technical difficulties during anastomosis, are needed to give further validation of RDN procedure in the receiver cohort.

## Data Availability

Anonymized full database is available on demand.

## Abbreviations

LDN: Living donor nephrectomy
RDN: Robot-assisted donor nephrectomy
HLDN: Hand-assisted laparoscopic nephrectomy
ESRF: End-stage renal failure
